# Supplementary material for: Extremely confined gap plasmon modes: when nonlocality matters
Source: Nat Commun. 2022 Jun 3;13:3105. doi: 10.1038/s41467-022-30737-2 (PMC9166740; doi:10.1038/s41467-022-30737-2)
Supplement: Supplementary file 1 — Supplementary Information [file 41467_2022_30737_MOESM1_ESM.pdf]

Extremely confined gap plasmon modes: when nonlocality matters — Supplementary Information

Sergejs Boroviks,[1,2] Zhan-Hong Lin,[2] Vladimir A. Zenin,[1] Mario Ziegler,[2] Andrea Dellith,[2] P. A. D. Gonçalves,[1] Christian Wolff,[1] Sergey I. Bozhevolnyi,[1,3] Jer-Shing Huang,[2,4,5,6] and N. Asger Mortensen[1,3]

[1]*Center for Nano Optics, University of Southern Denmark, Campusvej 55, DK-5230 Odense M, Denmark*
[2]*Leibniz Institute of Photonic Technology, Albert-Einstein Straße 9, 07745 Jena, Germany*
[3]*Danish Institute for Advanced Study, University of Southern Denmark, Campusvej 55, DK-5230 Odense M, Denmark*
[4]*Institute of Physical Chemistry and Abbe Center of Photonics, Friedrich–Schiller University Jena, Jena, Germany*
[5]*Research Center for Applied Sciences, Academia Sinica, 128 Sec. 2, Academia Road, Nankang District, 11529 Taipei, Taiwan*
[6]*Department of Electrophysics, National Chiao Tung University, 1001 University Road, 30010 Hsinchu, Taiwan*

(Dated: April 7, 2022)

S1. SAMPLE FABRICATION DETAILS

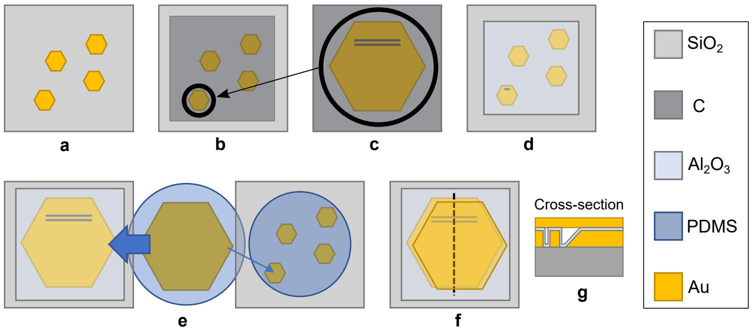

FIG. S1. **a** Synthesis of gold flakes on BK-7 glass substrate; **b** Coating sample with ~ 6 nm conductive carbon (C) layer for subsequent SEM and FIB milling; **c** Identification of a suitable gold flake sample on the substrate using optical and scanning electron microscopy, with subsequent milling of the coupling-element slits; **d** Removal of carbon layer and atomic-layer deposition (ALD) of aluminum oxide (Al_2O_3) layer; **e** Transfer of a thin gold flake from another substrate onto the milled structure using a polydimethylsiloxane (PDMS) stamp; **f** Top view and **g** cross-section of the final structure.

S2. MICROGRAPHS OF ALL FABRICATED SAMPLES

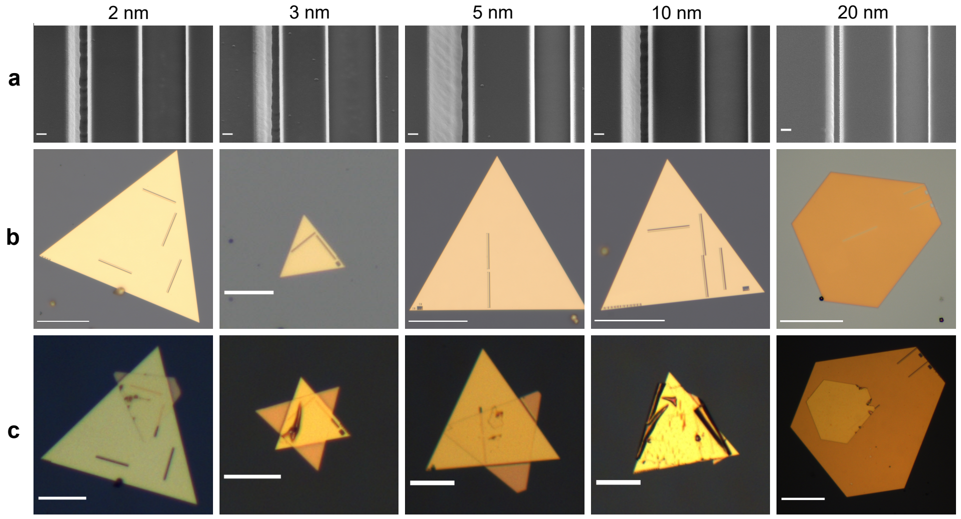

FIG. S2. Columns correspond to the samples with indicated nominal ALD layer thicknesses.**a** SEM close-up of coupling elements (scale bars: 100 nm); **b** optical micrographs before transfer step (scale bars: 30 µm); **c** optical micrographs of the fabricated samples, after transfer of the top flakes (scale bars: 30 µm).

S3. S-SNOM MEASUREMENTS: NEAR-FIELD MAPS OF ALL SAMPLES

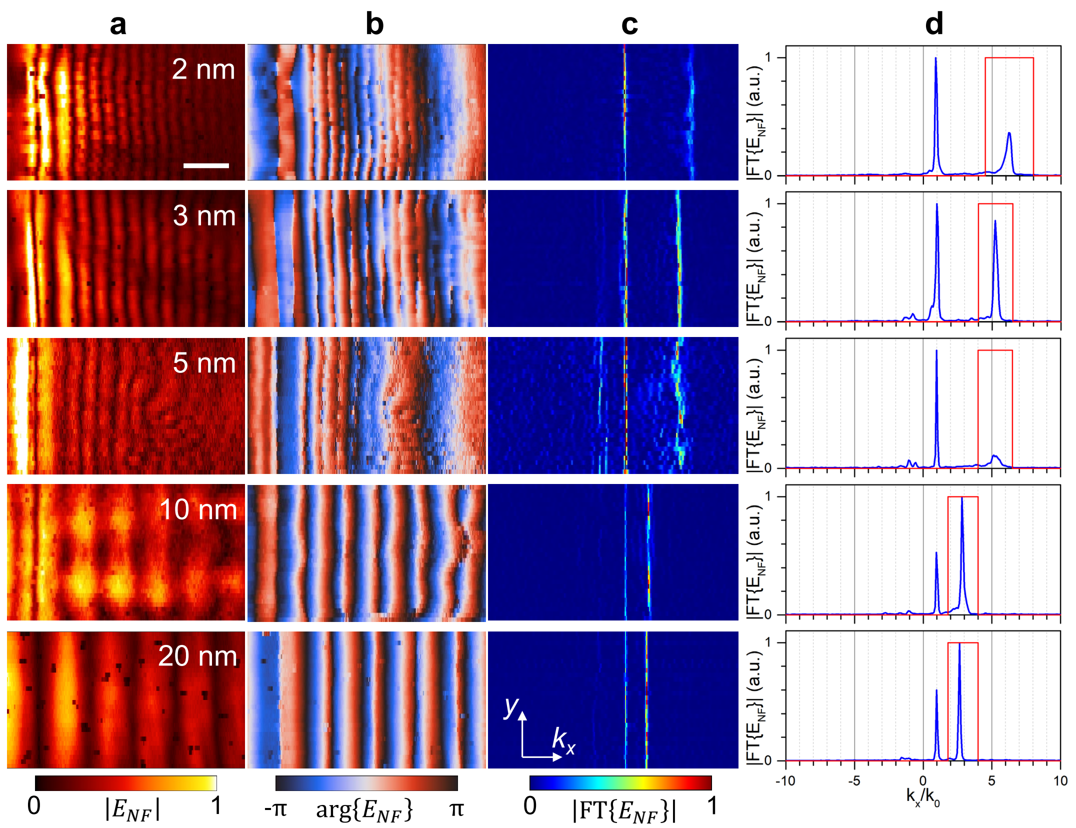

FIG. S3. **a** Near-field amplitude and **b** phase, measured for different samples with indicated alumina thickness. Scale bar: 1 µm. **c** Amplitude of the corresponding Fourier spectra and **d** its profile, averaged along y-axis. Red line indicate rectangular apodization function, applied for filtering GSP mode.

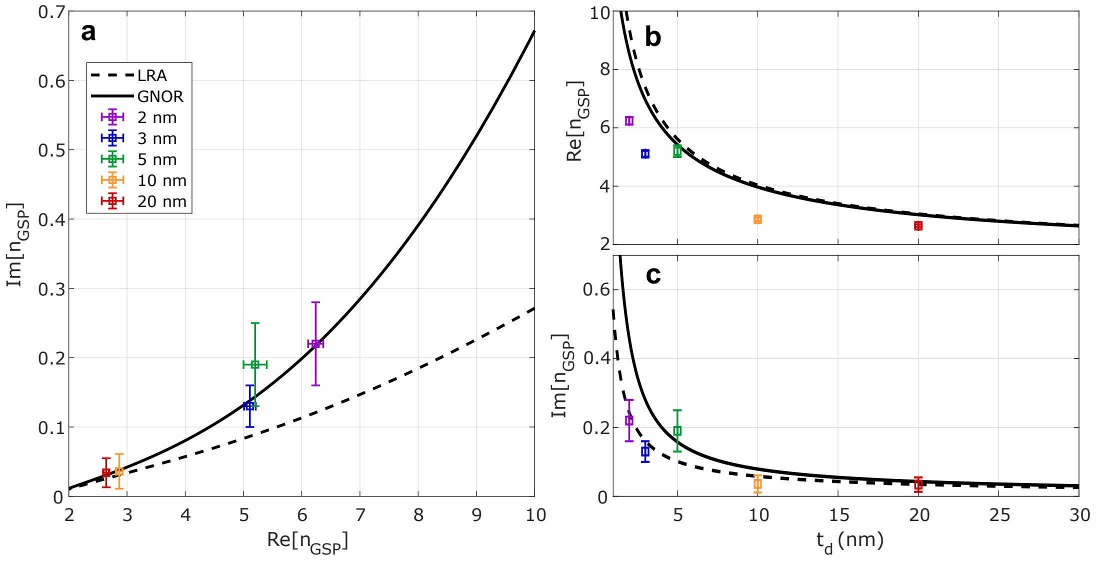

FIG. S4. Representation of effective-mode index n_{GSP}: experimentally obtained data points (squares with error bars) and dispersion curves calculated within the LRA (dashed lines) and GNOR (solid lines): **a** Parametric plot; **b** real and **c** imaginary part of the effective mode index versus nominal dielectric gap thickness t_{d}.

S4. ANALYSIS OF THE NEAR-FIELD DATA

Here we describe the procedure of processing measured near-field data. For all samples the scanned length and width was 10 and 3 microns, respectively, with the waveguide coupler being in the center. Although the top gold flake covers the waveguide coupler, hindering its easy identification via topography (Fig. S5a), it was possible to identify its position by two distinct lines in near-field amplitude (Fig. S5b), corresponding to the position of the major slit in the waveguide coupler (see inset in Fig. S5a). Next, we selected area outside of the coupler (green dashed line in Fig. 5b) and applied extended discrete Fourier transform (EDFT) [S1, S2] along the x-axis to identify propagating waves (Fig. S5d,e). The GSP mode appears as a pronounced peak with an effective mode index $k_x/k_0 > 2$ (around 5 for the alumina thickness of 3 nm, Fig. S5e). Then we filtered this mode with a reasonably wide rectangular apodization function (with a width of 3.5 for 2 nm alumina; 2.5 for 3 and 5 nm alumina; and 2.2 for 10 and 20 nm alumina). The result of Fourier filtering, E_{FT} (Fig. S5f,g) was then line-by-line fitted with a complex exponent function in order to precisely determine the real and imaginary part of n_{GSP} (Fig. S5h). The fitted field distribution, E_{fit}, and residuals can be found in Fig. S5i-j and k-l, correspondingly. The goodness of fitting for each line was determined as $1 - \frac{\int |E_{\mathrm{FT}} - E_{\mathrm{fit}}|^2 dx}{\int |E_{\mathrm{FT}}|^2 dx}$, reaching 1 for the perfect fit and 0 for no fit at all. For each alumina thickness there were 5-10 scans, done in different positions of the sample, resulting in 150-300 points with n_{GSP}. The weighted mean was then used to determine the average n_{GSP}, with weighted standard deviation used to determine the error bars for each alumina thickness, with the goodness of fitting used as a weight.

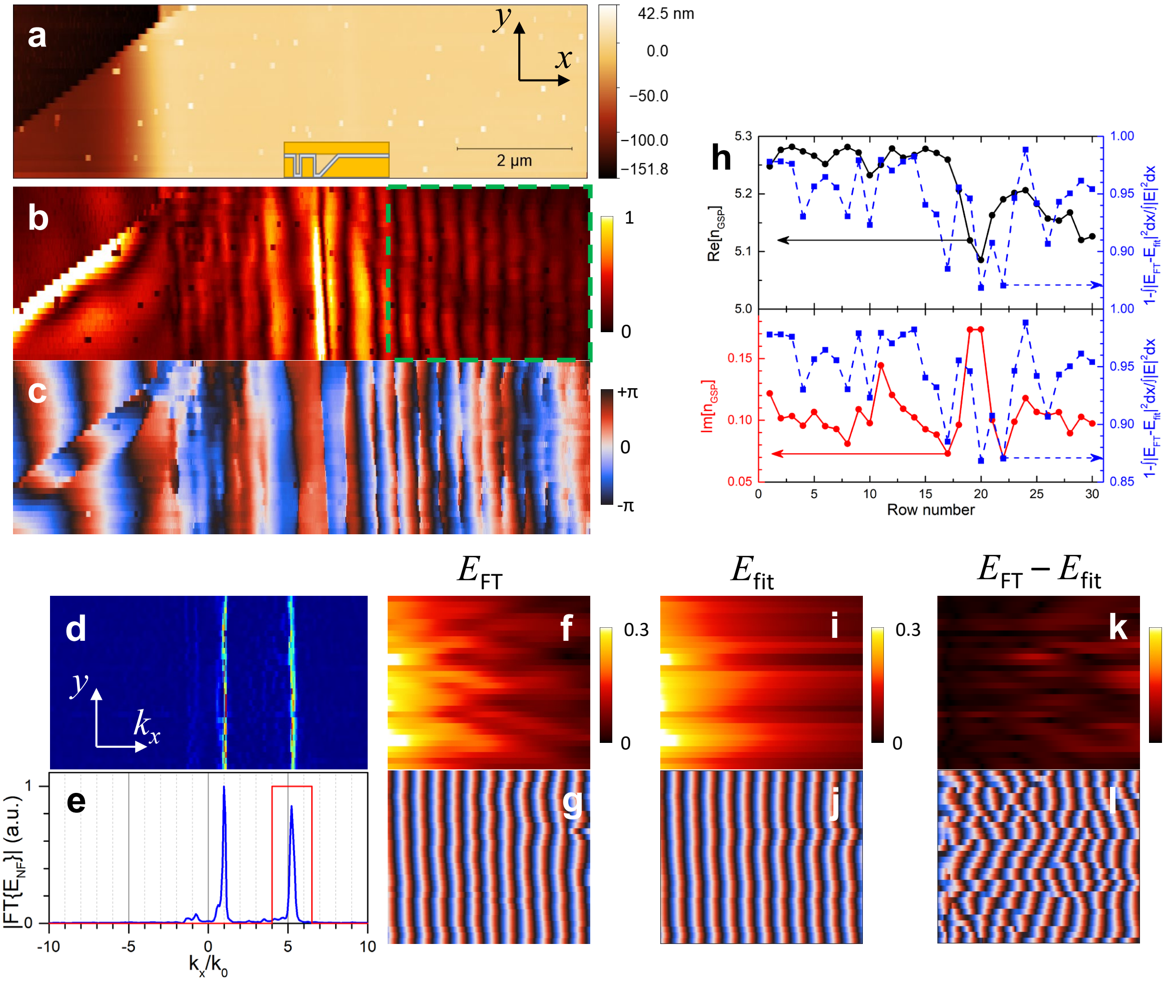

FIG. S5. Recorded **a** AFM topography and near-field **b** amplitude and **c** phase for the sample with 3 nm alumina. Inset in **a** shows an approximate x-axis position of the waveguide coupler. Dashed green frame in **b** indicate the area for Fourier analysis. **d** Amplitude of the Fourier transform of the recorded near-field along x-axis and **e** its y-axis average. Red line in **e** depict used rectangular apodization function, used to filter GSP mode. **f, g** Amplitude and phase of the near-field after Fourier filtering. **h** GSP effective mode index for each y-axis line, acquired by fitting the filtered field with a complex exponent. **i-l** Fitted field and residuals.

S5. FITTING THE DIFFUSION CONSTANT $\mathcal{D}$

Estimation of the diffusion constant $\mathcal{D}$ was performed by fitting the GNOR dispersion relation (eq. 1a) (with $\mathcal{D}$ being a free parameter) to the imaginary part of the experimentally obtained effective mode index n_{GSP} for the corresponding experimental values of real part of n_{GSP}. Since $\mathrm{Re}\{n_{\mathrm{GSP}}\}$ is nearly independent of $\mathcal{D}$ (see Fig. S4), this permits to make a fit to solely imaginary part, by considering only those thicknesses t_{d} which satisfy the experimental value of $\mathrm{Re}\{n_{\mathrm{GSP}}\}$.

Fig. S6 compares the result of our fit against the values reported in previous works, summarized in Table S1.

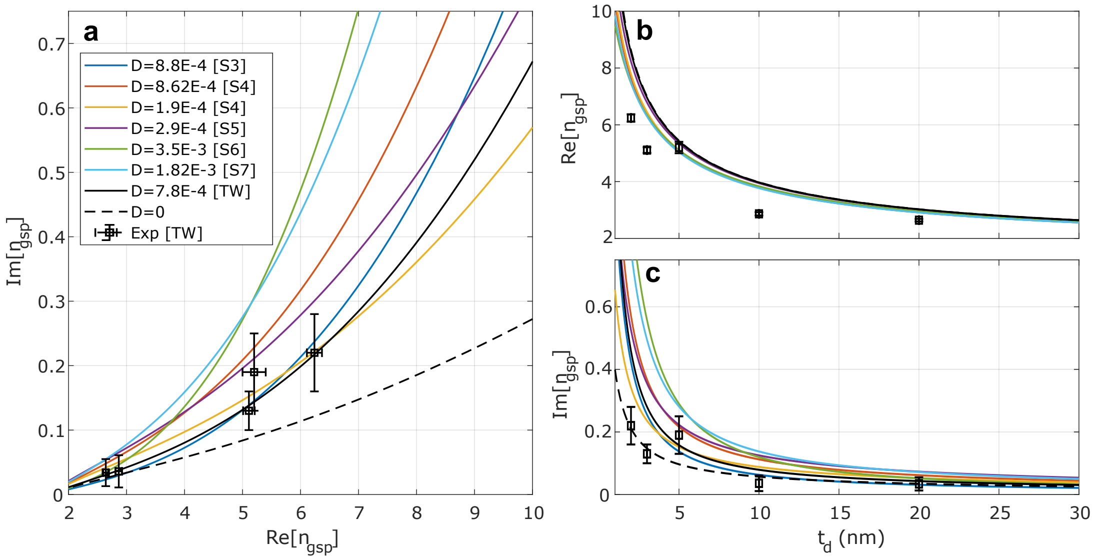

FIG. S6. Comparison of the parametric plots obtained with the diffusion constant $\mathcal{D}$ reported in literature and in this work [TW]: **a** Parametric plot; **b** real and **c** imaginary part of the effective mode index versus nominal dielectric gap thickness.

$\mathcal{D}$ (10^{-4} m^2 s^{-1})	v_{F} (10^6m s^{-1})	$\hbar\omega_{\mathrm{p}}$ (eV)	$\hbar\gamma_{\mathrm{p}}$ (meV)	Reference
8.8	1.4	8.99	26	[S3]
8.62	1.39	9.02	71	[S4]
1.9	1.39	9.02	71	[S4]
2.9	1.39	8.29	90	[S5]
35	1.39	8.55	18	[S6]
18.2	1.4	9	71	[S7]
7.85	1.39	8.29	47	[TW]

Table S1. GNOR parameters reported in previous works and this work [TW]: diffusion constant $\mathcal{D}$, Fermi velocity v_{F}, plasma frequency $\hbar\omega_{\mathrm{p}}$ and damping rate $\hbar\gamma_{\mathrm{p}}$.

S6. DEPENDENCE OF THE DISPERSION RELATION ON MATERIAL PARAMETERS ε_m AND ε_d

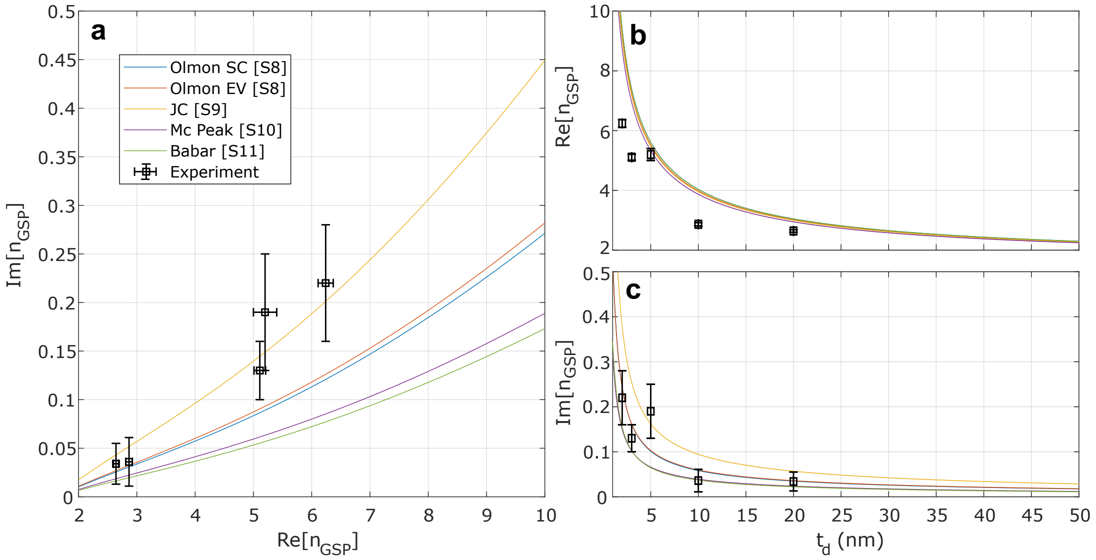

FIG. S7. Dispersion curves calculated within the LRA using various gold's permittivity data from literature [S8–S11] in comparison with experimentally-measured data points. **a** Parametric plot of the dispersion curves; **b** real and **c** imaginary part of the effective mode index versus nominal dielectric gap thickness.

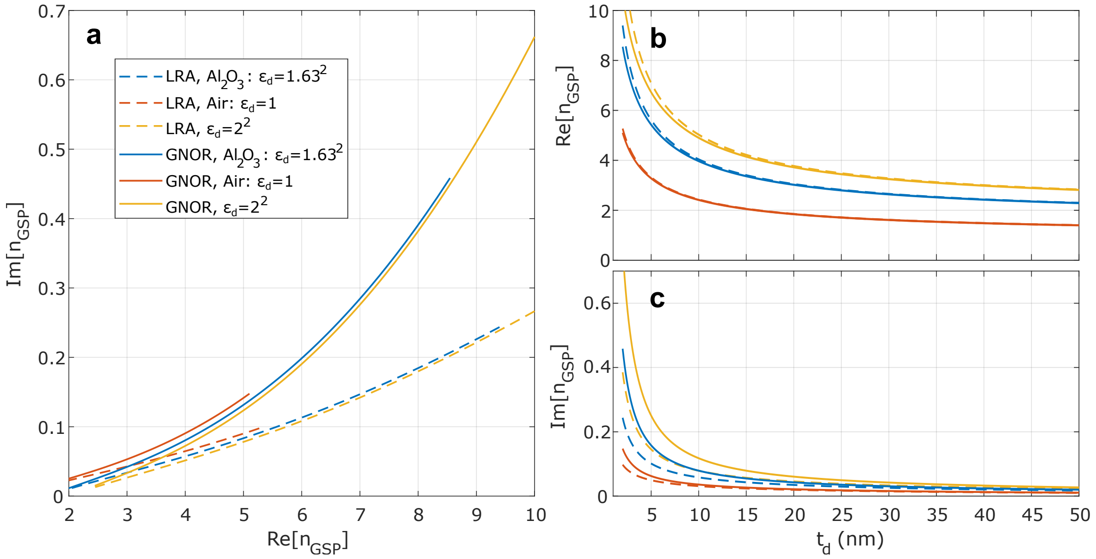

FIG. S8. Comparison of the dispersion curves calculated with LRA and GNOR using various values of dielectric constants of the material in the gap. **a** Parametric plot of the dispersion curves; **b** real and **c** imaginary part of the effective mode index versus nominal dielectric gap thickness.

S7. DISPERSION OF GSP MODES IN COMPARISON WITH SPP AT AIR-GOLD INTERFACE

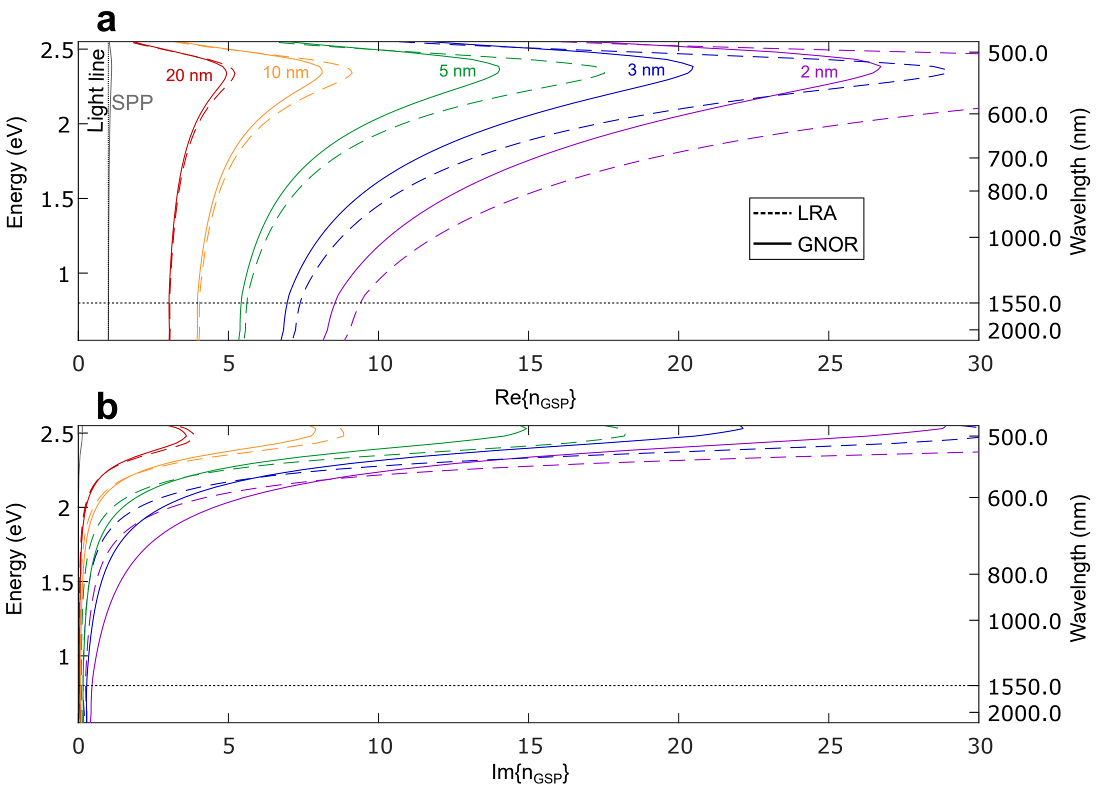

FIG. S9. Comparison of the GSP dispersion curves for the indicated dielectric gap thicknesses (2, 3, 5, 10 and 20 nm) calculated within LRA (dashed) and GNOR (solid) models in contrast to a bare SPP at air-gold interface. **a** Real and **b** imaginary parts of the dispersion curves for various dielectric gap thicknesses (color coded).

S8. INFLUENCE OF AN ADDITIONAL AIR VOID

One of the possible explanations for persistently smaller real part of the experimentally retrieved n_{GSP} (see Fig. S4b) could be existence of an additional air layer between dielectric ALD layer and upper gold flake. As mentioned in the main text, presence of such air void could be caused by imperfections in the sample fabrication, for example contamination during the transfer step or inhomogeneous adhesion of the upper gold flake to the ALD layer. This results in local variation of the gap thickness and effective permittivity of the dielectric layer, consequently broadening peak corresponding to n_{GSP} in the real part of k_x spectrum (see Fig. S3d). Here, we demonstrate implications of such a thin air layer for the dispersion relation and show compatibility of this assumption with the interpretation of our experimental results.

We suppose that the gap between metal flakes is composed of alumina layer (of nominal thickness t_{ALD} calculated by multiplying the number of ALD cycles by the average ALD alumina growth rate) and an additional layer of air (with thickness t_{air}) , see Fig. S10. This results in a lower effective dielectric constant of the material in the gap, which is calculated using the effective medium theory, according to the formula:

$$\varepsilon_{d,eff} = \frac{\varepsilon_{ALD}\varepsilon_{air}}{f_{air}\varepsilon_{ALD} + f_{ALD}\varepsilon_{air}} \tag{S1}$$

with $f_{air} = \frac{t_{air}}{t_{air}+t_{ALD}}$ and $f_{ALD} = \frac{t_{ALD}}{t_{air}+t_{ALD}}$ being air and alumina volume factors, correspondingly.

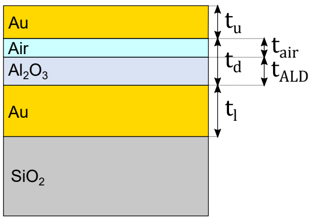

FIG. S10. Schematic illustration of the considered geometry with additional air void: dielectric gap of thickness t_d consists of an ALD layer of thickness t_{ALD} and an additional air layer of thickness t_{air}.

An additional air void results not only in reduced effective dielectric constant of the gap, but also in increased total gap size, which both leads to the reduced real and imaginary parts of the GSP effective mode index (as can be seen in Fig. S11, for both local and nonlocal models).However, when dispersion is plotted in the parametric graph (Fig. S11a), the addition of the air layer has negligible influence on the curve position, similarly to the negligible influence of dielectric constants of the material in the gap [Fig. S8a]. This allows to experimentally quantify the influence of non-local corrections without precise knowledge of gap composition.

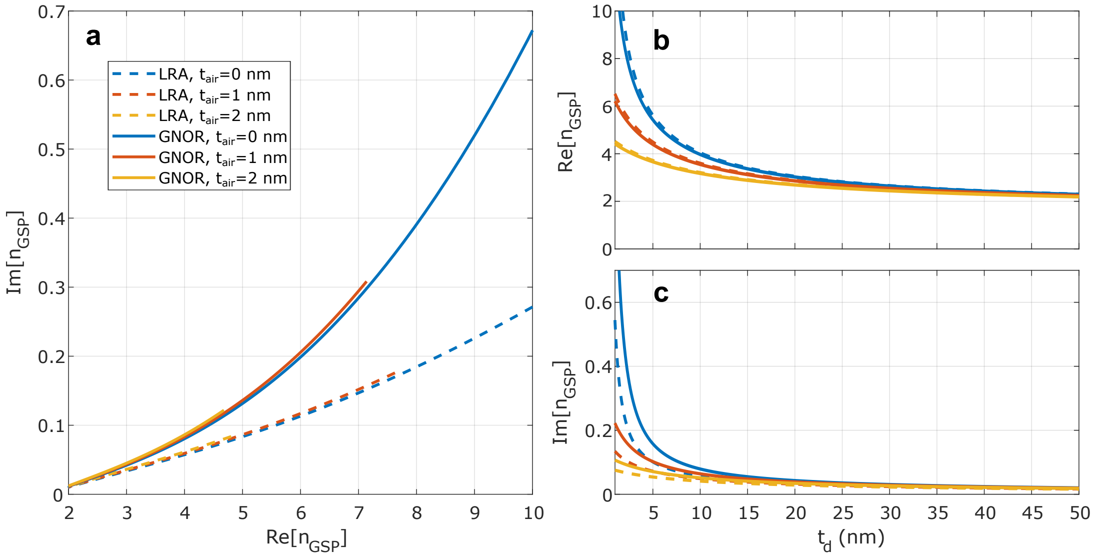

FIG. S11. Comparison of the dispersion curves calculated within LRA (dashed curves) and GNOR (solid curves) assuming different thicknesses of an additional air layer in the dielectric gap, with effective dielectric permittivity obtained using Eq. S1. **a** Parametric plot of the dispersion curves; **b** real and **c** imaginary parts of the effective mode index versus dielectric gap thickness.

The real part of the effective mode index n_{GSP} depends on both effective permittivity of the gap material $\varepsilon_{\mathrm{d,eff}}$ and gap thickness t_{d}. Since non-local corrections have negligible influence on the real part of n_{GSP} for our range of gaps (Fig. S11), one can find pairs of $\varepsilon_{\mathrm{d,eff}}$ and t_{d}, resulting in the same Re$\{n_{\mathrm{GSP}}\}$ as was experimentally measured (black solid lines in Fig. S12, with gray margins corresponding to the experimentally measured error bar of Re$\{n_{\mathrm{GSP}}\}$). Next, assuming the gap composition as ALD alumina layer of thickness t_{ALD} with additional air layer of thickness t_{air} (see previous section), such that $t_{\mathrm{d}} = t_{\mathrm{air}} + t_{\mathrm{ALD}}$, one can plot effective permittivity of the gap, $\varepsilon_{\mathrm{d,eff}}$ vs. air layer thickness, t_{air} (dashed colored lines in Fig. S12). The intersection of these curves with previously defined black solid lines provides estimation of the additional air layer thickness in measured samples, which was found to be (see table I).

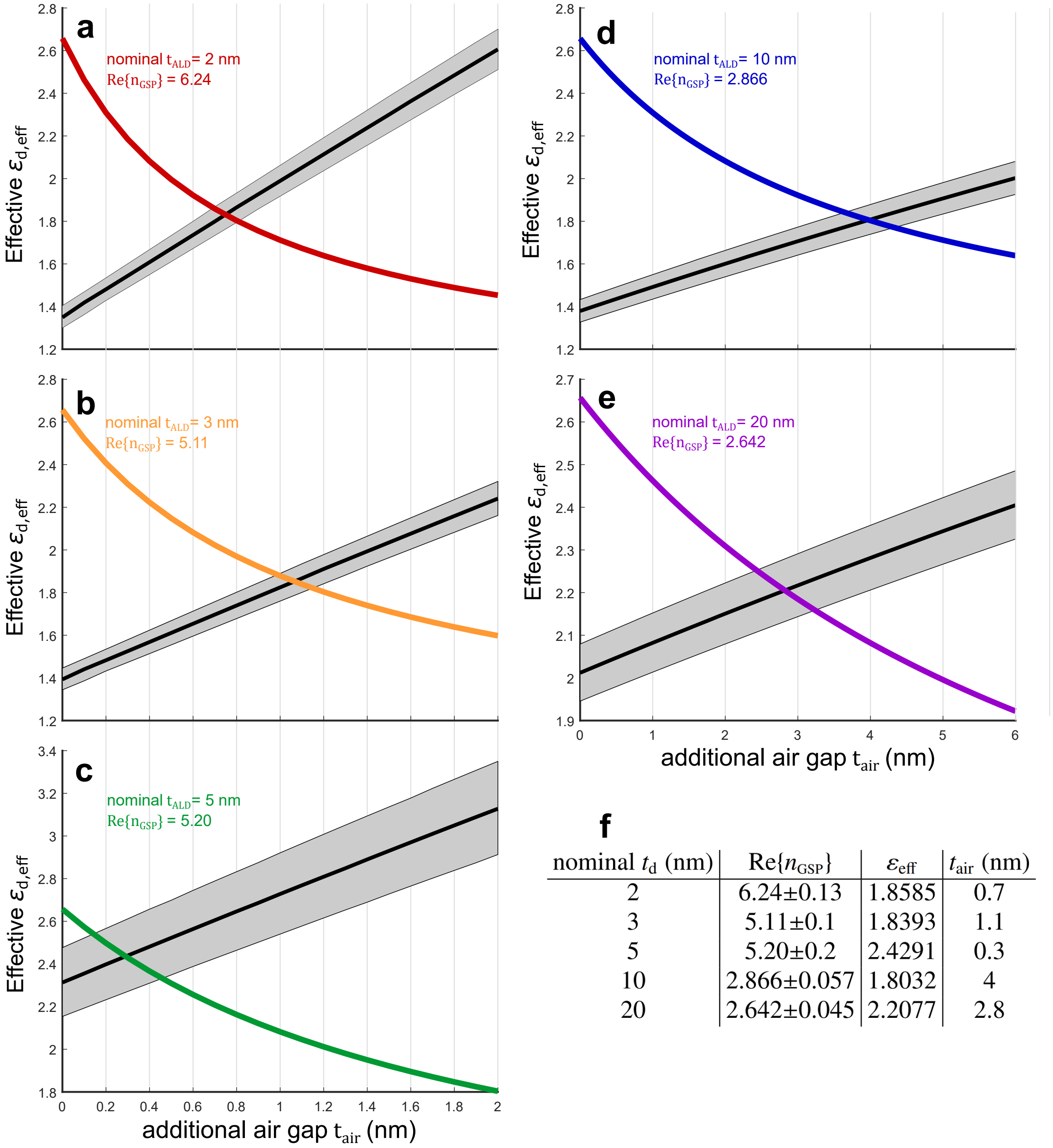

nominal t_{d} (nm)	Re$\{n_{\mathrm{GSP}}\}$	$\varepsilon_{\mathrm{eff}}$	t_{air} (nm)
2	6.24±0.13	1.8585	0.7
3	5.11±0.1	1.8393	1.1
5	5.20±0.2	2.4291	0.3
10	2.866±0.057	1.8032	4
20	2.642±0.045	2.2077	2.8

FIG. S12. **a-e** Estimation of thickness of an additional air void in the 5 considered samples with varying nominal dielectric gap thickness and **f** summarizing table.

S9. NUMERICAL SIMULATIONS: OPTIMIZATION OF THE COUPLER DESIGN

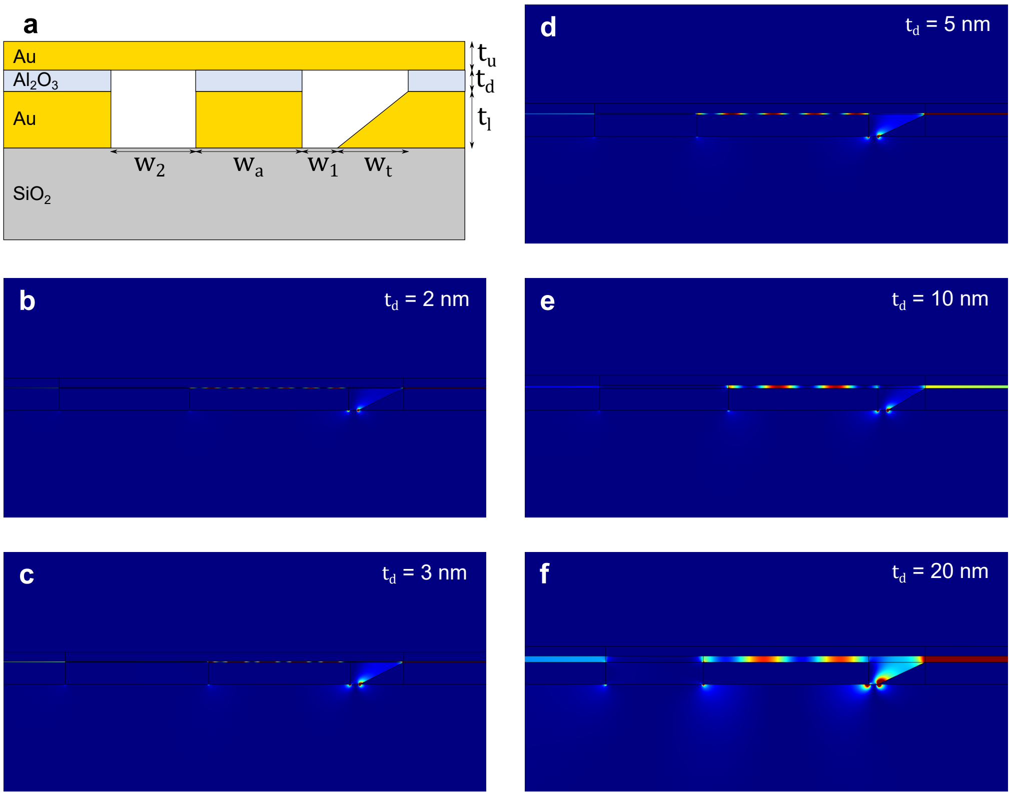

FIG. S13. **a** Geometry of the tapered waveguide coupler **b-f** Pseudo-color images of the simulated vertical component of the electric field E_z in the vicinity of tapered couplers for different thicknesses of the gaps in metal–dielectric–metal (MDM) planar waveguides.

t_d (nm)	w_t (nm)	w_1 (nm)	w_a (nm)	w_2 (nm)	Re$\{n_{gsp}\}$	η_c
2	145	30	505	415	6.24±0.13	10.7%
3	140	30	450	455	5.11±0.1	11.4%
5	150	30	545	325	5.20±0.2	11.3%
10	120	30	475	410	2.866±0.057	12.2%
20	150	30	525	310	2.642±0.045	13.3%

Table S2. Optimized geometric parameters of the tapered couplers for the MDM waveguides with various thicknesses of dielectric gap t_d.

S10. FEIBELMAN d-PARAMETERS

Modified boundary conditions

Feibelman d-parameters can be incorporated into the conventional boundary conditions, by considering surface (normal) polarization $\boldsymbol{P}(\boldsymbol{r}) = \boldsymbol{\pi}(\boldsymbol{r})\delta(\boldsymbol{r} - \boldsymbol{r}_{\delta\Omega})$ and surface (tangential) current $\boldsymbol{J}(\boldsymbol{r}) = \boldsymbol{K}(\boldsymbol{r})\delta(\boldsymbol{r} - \boldsymbol{r}_{\delta\Omega})$ which are driven by the discontinuities of the $\boldsymbol{E}$ and $\boldsymbol{D}$ fields [S12]:

$$\boldsymbol{\pi} \equiv \varepsilon_0 d_\perp \left[\hat{\boldsymbol{n}} \cdot (\boldsymbol{E}_\mathrm{d} - \boldsymbol{E}_\mathrm{m})\right] \hat{\boldsymbol{n}}, \tag{S2a}$$

$$\boldsymbol{K} \equiv i\omega d_\parallel \left[\hat{\boldsymbol{n}} \times (\boldsymbol{D}_\mathrm{d} - \boldsymbol{D}_\mathrm{m}) \times \hat{\boldsymbol{n}}\right]. \tag{S2b}$$

The boundary conditions can be stated via the parallel and perpendicular components of the fields as:

$$\boldsymbol{E}_{\mathrm{d}\parallel} - \boldsymbol{E}_{\mathrm{m}\parallel} = -d_\perp \boldsymbol{\nabla}_\parallel \left(E_{\mathrm{d}\perp} - E_{\mathrm{m}\perp}\right), \tag{S3a}$$

$$\boldsymbol{H}_{\mathrm{d}\parallel} - \boldsymbol{H}_{\mathrm{m}\parallel} = i\omega d_\parallel \left(\boldsymbol{D}_{\mathrm{d}\parallel} - \boldsymbol{D}_{\mathrm{m}\parallel}\right) \times \hat{\boldsymbol{n}}. \tag{S3b}$$

SPP dispersion at a planar MD interface with Feibelman d-parameter correction

The d-parameter-corrected reflection coefficient for p-polarized waves associated with a planar dielectric–metal interface reads [S12–S14]

$$r_p^\mathrm{DM} = \frac{\varepsilon_\mathrm{m} k_{z,\mathrm{d}} - \varepsilon_\mathrm{d} k_{z,\mathrm{m}} + (\varepsilon_\mathrm{m} - \varepsilon_\mathrm{d})[\mathrm{i}q^2 d_\perp - \mathrm{i}k_{z,\mathrm{d}}k_{z,\mathrm{m}}d_\parallel]}{\varepsilon_\mathrm{m} k_{z,\mathrm{d}} + \varepsilon_\mathrm{d} k_{z,\mathrm{m}} - (\varepsilon_\mathrm{m} - \varepsilon_\mathrm{d})[\mathrm{i}q^2 d_\perp + \mathrm{i}k_{z,\mathrm{d}}k_{z,\mathrm{m}}d_\parallel]}, \tag{S4}$$

where $k_{z,j} = \sqrt{\frac{\omega^2}{c^2}\epsilon_j - q^2}$, with $j \in \{\mathrm{d}, \mathrm{m}\}$ and subject to $\mathrm{Im}\, k_{z,j} \geq 0$.

The surface plasmon polariton (SPP) dispersion relation then follows from the poles of r_p^DM, yielding [S12, S13]

$$\varepsilon_\mathrm{m}\kappa_\mathrm{d} + \varepsilon_\mathrm{d}\kappa_\mathrm{m} - (\varepsilon_\mathrm{m} - \varepsilon_\mathrm{d})\left[q^2 d_\perp - \kappa_\mathrm{d}\kappa_\mathrm{m} d_\parallel\right] = 0 \tag{S5}$$

where we have defined $\kappa_j \equiv -\mathrm{i}k_{z,j} = \sqrt{q^2 - \epsilon_j \frac{\omega^2}{c^2}}$.

SPP dispersion for a MDM structure with Feibelman d-parameter correction

Let us now consider a vertical heterostructure $\epsilon_1|\epsilon_2|\epsilon_3$. In this case, the poles of the corresponding scattering amplitudes (i.e., the reflection and transmission coefficients) are given by $1 - r^{21}r^{23}\exp(2\mathrm{i}k_{z,2}t) = 0$. Here, t denotes the thickness of the inner slab (ϵ_2) and r^{kl} stand for the reflection coefficients describing the reflection of electromagnetic waves from a planar interface defining the boundary between "medium k" and "medium l", with incidence coming from the former [S15]. Hence, the dispersion relation for SPP modes in MDM structures are given by ($\epsilon_1, \epsilon_3 \to \varepsilon_\mathrm{m}$ and $\epsilon_2 \to \varepsilon_\mathrm{d}$)

$$1 - r_p^\mathrm{DM} r_p^\mathrm{DM} \mathrm{e}^{2\mathrm{i}k_{z,2}t} = 0, \tag{S6a}$$

$$\Rightarrow r_p^\mathrm{DM} \mathrm{e}^{\mathrm{i}k_{z,2}t} = \pm 1. \tag{S6b}$$

Combining Eqs. (S4) and (S6b), we obtain the sought-after implicit condition for the dispersion relation of the two coupled SPPs modes (with opposite symmetries) supported by a MDM structure:

$$\tanh\left(\frac{\kappa_\mathrm{d} t}{2}\right) = -\frac{\varepsilon_\mathrm{d}\kappa_\mathrm{m} - (\varepsilon_\mathrm{m} - \varepsilon_\mathrm{d})q^2 d_\perp}{\varepsilon_\mathrm{m}\kappa_\mathrm{d} + (\varepsilon_\mathrm{m} - \varepsilon_\mathrm{d})\kappa_\mathrm{d}\kappa_\mathrm{m} d_\parallel}, \tag{S7a}$$

$$\coth\left(\frac{\kappa_\mathrm{d} t}{2}\right) = -\frac{\varepsilon_\mathrm{d}\kappa_\mathrm{m} - (\varepsilon_\mathrm{m} - \varepsilon_\mathrm{d})q^2 d_\perp}{\varepsilon_\mathrm{m}\kappa_\mathrm{d} + (\varepsilon_\mathrm{m} - \varepsilon_\mathrm{d})\kappa_\mathrm{d}\kappa_\mathrm{m} d_\parallel}. \tag{S7b}$$

Notice that the classical MDM results are recovered in the $d_{\perp,\parallel} \to 0$ limit, and the uncoupled, single-interface SPP dispersion is reinstated in the limit of an extremely thick inner slab, i.e., in the $t \to \infty$ limit.

In the nonretarded regime, Eqs. (S7) become:

$$\tanh\left(\frac{qt}{2}\right) = -\frac{\varepsilon_{\rm d} - (\varepsilon_{\rm m} - \varepsilon_{\rm d})qd_\perp}{\varepsilon_{\rm m} + (\varepsilon_{\rm m} - \varepsilon_{\rm d})qd_\parallel}, \tag{S8a}$$

$$\coth\left(\frac{qt}{2}\right) = -\frac{\varepsilon_{\rm d} - (\varepsilon_{\rm m} - \varepsilon_{\rm d})qd_\perp}{\varepsilon_{\rm m} + (\varepsilon_{\rm m} - \varepsilon_{\rm d})qd_\parallel}. \tag{S8b}$$

This result agrees well with the previously reported [S16].

Relation to hydrodynamic treatment

Returning to Eqs. (S7) for $d_\parallel = 0$ we get

$$\tanh\left(\frac{\kappa_{\rm d}\, t}{2}\right) = -\frac{\varepsilon_{\rm d}\kappa_{\rm m}}{\varepsilon_{\rm m}\kappa_{\rm d}}\left[1 - \frac{\varepsilon_{\rm m} - \varepsilon_{\rm d}}{\varepsilon_{\rm d}}\frac{q^2 d_\perp}{\kappa_{\rm m}}\right], \tag{S9a}$$

$$\coth\left(\frac{\kappa_{\rm d}\, t}{2}\right) = -\frac{\varepsilon_{\rm d}\kappa_{\rm m}}{\varepsilon_{\rm m}\kappa_{\rm d}}\left[1 - \frac{\varepsilon_{\rm m} - \varepsilon_{\rm d}}{\varepsilon_{\rm d}}\frac{q^2 d_\perp}{\kappa_{\rm m}}\right], \tag{S9b}$$

so that in the context of Ref. S17 we have $\delta_{\rm nl} = -\frac{\varepsilon_{\rm m}-\varepsilon_{\rm d}}{\varepsilon_{\rm d}}\frac{q^2 d_\perp}{\kappa_{\rm m}} \simeq -\frac{\varepsilon_{\rm m}-\varepsilon_{\rm d}}{\varepsilon_{\rm d}} q d_\perp$.

SUPPLEMENTARY REFERENCES

[S1] V. Liepins, "Extended fourier analysis of signals," (2019), arXiv:1303.2033 [cs.DS].
[S2] "Mathworks file exchange: Extended dft version 1.46.2.0 (23.1 kb) by vilnis liepins," `https://www.mathworks.com/matlabcentral/fileexchange/11020-extended-dft`, accessed: 31.08.2021.
[S3] H. Shen, L. Chen, L. Ferrari, M.-H. Lin, N. A. Mortensen, S. Gwo, and Z. Liu, Nano Letters **17**, 2234 (2017).
[S4] S. Raza, M. Wubs, S. I. Bozhevolnyi, and N. A. Mortensen, Opt. Lett. **40**, 839 (2015).
[S5] M. K. Dezfouli, C. Tserkezis, N. A. Mortensen, and S. Hughes, Optica **4**, 1503 (2017).
[S6] M. G. Blaber, M. D. Arnold, and M. J. Ford, The Journal of Physical Chemistry C **113**, 3041 (2009).
[S7] C. David and J. Christensen, Applied Physics Letters **110**, 261110 (2017).
[S8] R. L. Olmon, B. Slovick, T. W. Johnson, D. Shelton, S.-H. Oh, G. D. Boreman, and M. B. Raschke, Phys. Rev. B **86**, 235147 (2012).
[S9] P. B. Johnson and R. W. Christy, Phys. Rev. B **6**, 4370 (1972).
[S10] K. M. McPeak, S. V. Jayanti, S. J. P. Kress, S. Meyer, S. Iotti, A. Rossinelli, and D. J. Norris, ACS Photonics **2**, 326 (2015).
[S11] S. Babar and J. H. Weaver, Appl. Opt. **54**, 477 (2015).
[S12] P. A. D. Gonçalves, T. Christensen, N. Rivera, A.-P. Jauho, N. A. Mortensen, and M. Soljačić, Nat. Commun. **11**, 366 (2020).
[S13] P. A. D. Gonçalves, *Plasmonics and Light–Matter Interactions in Two-Dimensional Materials and in Metal Nanostructures: Classical and Quantum Considerations* (Springer Nature, 2020).
[S14] A. Liebsch, *Electronic Excitations at Metal Surfaces* (Springer, Boston, 1997).
[S15] It should be noted that—and contrary to the classical case—for d-parameter-corrected reflection coefficients, in general, one has $r^{kl} \neq -r^{lk}$.
[S16] T. Christensen, W. Yan, A.-P. Jauho, M. Soljačić, and N. A. Mortensen, Phys. Rev. Lett. **118**, 157402 (2017).
[S17] S. Raza, T. Christensen, M. Wubs, S. I. Bozhevolnyi, and N. A. Mortensen, Phys. Rev. B **88**, 115401 (2013).
